# Maladaptive rumination moderates the effects of written emotional disclosure on ambulatory blood pressure levels in females

**DOI:** 10.1080/21642850.2014.973881

**Published:** 2014-10-29

**Authors:** Daryl B. O'Connor, Laura Ashley, Fiona Jones, Eamonn Ferguson

**Affiliations:** ^a^ Institute of Psychological Sciences, University of Leeds, Leeds, UK; ^b^ School of Social, Psychological & Communication Sciences, Leeds Beckett University, Leeds, UK; ^c^ Department of Psychology, University of Nottingham, Nottingham, UK

**Keywords:** stress, personality, coping

## Abstract

Written emotional disclosure (WED) has beneficial effects on health outcomes. However, its effectiveness is influenced by a number of variables. This exploratory study tested whether trait rumination, which comprises brooding, a maladaptive component, and reflection, an adaptive component, moderated the effects of WED on ambulatory blood pressure (ABP) in female participants.

Fifty-two participants were randomized to write about their most stressful/traumatic life experience(s) or non-emotive topics, for 20 minutes, on 3 consecutive days. Two weeks and 14 weeks later, ABP was recorded over a single day.

Using hierarchical linear modelling, an effect of condition was found at 2 weeks but not at 14 weeks indicating that higher levels of ABP were observed following WED. There was also a significant condition by brooding interaction at two weeks such that higher ABP was observed in low brooders in the WED condition compared with low brooders in the control condition. However, within the WED condition, the lowest ABP was exhibited by participants high in brooding.

The findings indicated that WED led to short-lived increases in ABP which disappeared in the medium term. Researchers ought to build upon this exploratory study and investigate further the potential moderating role of brooding within WED. Individual differences in brooding may account for (some of) the mixed and inconsistent findings in past WED research.

## Introduction

Nearly 30 years ago James Pennebaker introduced the written emotional disclosure (WED) paradigm in a preliminary investigation to establish whether writing about traumatic events could influence long- and short-term indicators of health (Pennebaker & Beall, 1986). In this paradigm participants are normally asked to write about their deepest thoughts and emotions about a stressful or traumatic life experience; typically for 15–20 minutes on 3 or 4 consecutive days. The beneficial effects of the disclosure of stressful or traumatic experiences on numerous health outcomes have received considerable support (Frisina, Borod, & Lepore, 2004: Frattaroli, 2006; Smyth, Stone, Hurewitz, & Kaell, 1999) with Frattaroli reporting an average *r*-effect size of .075. For example, previous research has found the intervention to produce clinically significant improvements in lung function in asthmatic patients, enhanced immune functioning in healthy participants and HIV patients as well as increases in implicit self-esteem (O'Connor et al., 2011; Petrie, Fontanilla, Thomas, Booth, & Pennebaker, 2004; Smyth et al. 1999). Of relevance to the current study, a number of experimental studies have explored the effects of emotional disclosure on blood pressure (BP) but these have resulted in mixed findings and/or utilized verbal disclosure methods (Davidson et al., 2002; Lepore, Ragan, & Jones, 2000; O'Connor & Ashley, 2008; Pennebaker, Hughes, & O'Heeron, 1987). A notable exception is a study by Beckwith McGuire, Greenberg, and Gevirtz (2005) that found WED had the capacity to reduce resting BP in a sample of borderline hypertensives.

Nevertheless, a relatively large number of studies have failed to find positive results of the WED intervention (Frattaroli, 2006). This is not altogether unsurprising given that more than 200 studies have been published since 1986, but few if any have adopted identical designs. Moreover, numerous theories have been proposed to account for the positive effects of emotional disclosure (when they are observed) with varying degrees of evidence presented (for detailed discussion, see Frattaroli, 2006; Lepore & Smyth, 2002; Sloan & Marx, 2004). These theories include inhibition theory, cognitive processing theory, self-regulation theory and exposure theory. Therefore, it is highly unlikely that a single theoretical process explains the positive effects of WED.

It has also been suggested that the effectiveness of the intervention is influenced by a number of variables and that the boundary conditions of expressive writing need to be explored (Ashley, O'Connor, & Jones, 2011; Frattaroli, 2006; O'Connor & Ashley, 2008; Smyth & Pennebaker, 2008). Individual differences have been highlighted as one such boundary condition (Sloan, Marx, Epstein, & Dobbs, 2008). It has been argued that one way interventions such as WED are believed to operate is by encouraging participants to confront negative thoughts and emotions and to challenge hopeless and irrational cognitions (Nolen-Hoeksema, Wisco, & Lyubomirsky, 2008). As such, individuals who tend to focus on negative thoughts may benefit from interventions such as WED that help them to confront and challenge such thoughts and emotions (Sloan et al., 2008). Therefore, the current exploratory study examined the role of trait rumination as a moderator of the effects of WED.

Trait rumination is defined as a style of coping with negative and distressing events whereby the individual repeatedly and passively focuses on the symptoms of distress and their causes and consequences (Nolen-Hoeksema et al., 2008). Evidence suggests that rumination is best conceptualized as comprising brooding, a maladaptive component, and reflection, an adaptive component (Treynor, Gonzalez, & Nolen-Hoeksema, 2003). Once triggered, brooding is characterized by repeated negative thoughts about one's current situation and a passive focus on failure and hopelessness with little regard for active problem-solving. In contrast, reflective pondering is characterized by a determined focus inward to reflect on the situation in order to facilitate active problem-solving to reduce one's depressive symptoms. Indeed individuals with a brooding response tendency have been found to be at greater risk of experiencing depressive disorders, anxiety, binge eating and self-harm (Nolen-Hoeksema et al., 2008).

In the context of WED, a recent study by Sloan et al. (2008) demonstrated that individuals high and low in brooding responded differently to WED. Participants with a high brooding ruminative tendency were found to benefit from writing and reported lower depression symptoms at follow-up compared to those with a low brooding tendency. They also found that the WED paradigm had no significant beneficial effects among individuals with a reflective pondering response style. The authors argued that interventions such as WED may help overcome brooding ruminative response tendencies because they encourage participants to confront negative thoughts and emotions and to challenge hopeless and irrational cognitions rather than passively replaying or accepting them.

This pattern of findings and interpretation is consistent with personality theories, suggesting that traits not only influence people's situational preferences (Davis et al., 1999; Ferguson, 2013) but also their reactions to them with these reactions potentially reflecting a degree of trait change (Roberts & Jackson, 2008). An aspect of these models is the dynamic interplay between context, trait and biology (Roberts & Jackson, 2008). Therefore, this exploratory study aimed to extend previous findings by investigating if this potential beneficial effect of WED for rumination on consciously expressed emotions and cognitions was also observed for physiological reactions to WED. As outlined earlier, Beckwith McGuire and colleagues have found WED to reduce resting BP in a sample of borderline hypertensives 4 weeks after writing, although, the effect was not maintained at 4-month follow-up. Instead, these authors found that anger-in (i.e. the extent to which an individual does *not* express his/her anger in anger-arousing situations) moderated the WED effects, such that participants high in anger-in exhibited lower diastolic blood pressure (DBP) four months later, whereas, DBP increased in participants low in anger-in.

### The current study

This study sought to extend past research in two important ways. First, we wanted to examine the effects of WED on BP, an important health outcome, but also to utilize ambulatory blood pressure (ABP) monitoring techniques that allow for multiple sampling over a single day (Beckwith-McGuire et al., 2005; O'Connor & Ashley, 2008). This approach also overcomes problems associated with so-called white coat hypertension where participants can exhibit elevated BP in clinical/experimental settings and it can generate a large number of observations in real-world contexts, which in turn increase ecological validity. Second, we were keen to harness innovative multi-level modelling procedures that allow for the modelling of within-person variation (i.e. 30 minutes ABP readings throughout a day) together with between-person variability (i.e. writing condition and brooding score). Therefore, the current study, using a multi-level design, examined the extent to which brooding and reflection moderated the effects of WED on ABP in healthy participants during a working day; 2 weeks and 14 weeks following the intervention (while controlling for baseline BP levels and other potential confounders). The two-week follow-up was chosen as Smyth et al. (1999) have shown clinically significant effects of writing on objective health outcomes two weeks post-intervention in asthmatic patients. The 14-week follow-up assessment was chosen because we wanted to investigate whether any observed effects carried through into the medium term (i.e. 3–4 months post writing) similar to Beckwith McGuire et al.'s study. Given the mixed findings previously discussed and the exploratory nature of the study, we did not generate directional hypotheses. Instead, we hypothesized that there would be a main effect of writing condition on ABP levels at follow-up. We also predicted a significant writing condition × brooding interaction such that ABP levels would be different following WED in participants high and low in brooding.

## Method

### Design and participants

Seventy-two undergraduate students (62 females) were randomized to either a written emotion disclosure (WED; *n* = 36) or a control writing condition (*n *= 36). Five participants later withdrew or were lost to contact (two WED and three control), and for two participants (one WED and one control) baseline resting BP data were lost due to equipment failure. Exclusion criteria were: (1) being under 18 years, (2) suffering psychological illness (e.g. depression), (3) having diabetes, co-morbid heart disease or history of heart disease, (4) taking medication that affects cardiovascular activity (e.g. beta blockers) and (5) being pregnant. Students received course ‘participation credits’ for taking part. All students at our institution have the opportunity to take part in this scheme; however, it is not mandatory. Male participants and/or smokers (*n *= 13) were not included in the current analyses due to the established gender differences in cardiovascular disease aetiology and effects of smoking status on BP. The final sample consisted of 52 females (*n *= 25 in the WED condition, *n *= 27 in the control condition and yielded 2484 observations (including < 1% missing data)^1^. The mean age = 18.37 years (range 18–20 years), the mean body mass index (BMI) = 22.30 and 85.1% of the sample were Caucasian.

### Writing conditions

*Emotional disclosure*: Following typical Pennebaker-style disclosure instructions (Pennebaker & Beall, 1986), participants assigned to the WED condition were urged to “really let go” and write about their “very deepest emotions and thoughts” about stressful or traumatic experiences. Participants were free to write about any such experiences, and to write about the same experience repeatedly, or a number of different experiences. *Control*: As is typical in WED studies (Ashley, O'Connor, & Jones, 2013), control participants were asked to write objectively, without reference to their emotions and opinions, about their minute-by-minute activities on different days (e.g. yesterday and tomorrow).

### Measures

#### Sociodemographic and health information

A questionnaire assessing gender, age, ethnicity, parental hypertension and smoker status was completed at baseline. Participants’ height (metres) and weight (kilograms) were also measured in the laboratory at baseline, in order to calculate their BMI (weight/height^2^).

#### Brooding and reflection

Trait rumination was assessed using the 10-item version of the Ruminative Responses Scale (RRS) (Treynor et al., 2003). This measure comprises two 5-item subscales assessing brooding and reflection. The subscales were not significantly correlated in this study (*r* = 24, *p* = .08) and the internal reliability for both subscales in the current sample was good (Cronbach's *α* = > .70).

### Blood pressure

All BP measures were taken using SpaceLabs 90207 monitors (SpaceLabs, Redmond, Washington, DC, USA). Resting BP was measured in the laboratory at baseline (following customization) using their non-dominant arm while participants were still and seated in an upright position. Three BP measures were taken, two minutes apart, and the average of these was used. Acute factors affecting BP were controlled following BP measurement guidelines by Shapiro et al. (1996). Participants were asked to abstain from smoking, caffeine and large meals for 3 hours, and from alcohol for 12 hours, before the laboratory visit, and to verbally verify that they had adhered to this. In order to calibrate the BP monitor and allow participants to adapt to it, it was fitted at the start of the session and two measures were immediately taken one minute apart (and discarded). To enable participants to acclimatize to the laboratory, and to control for physical activity prior to the session, BP measures were taken after a 20-minute rest period during which participants were left alone and asked to relax while quietly browsing magazines.

ABP was assessed 2 weeks and 14 weeks post-writing. Participants wore a monitor for 12 hours (from 0900 h to 2100 h) on one weekday. Participants were asked to avoid formal exercise during monitoring and to remain as motionless as possible with the cuffed arm by their side during readings. The monitor was programmed to automatically inflate the arm cuff and, as is typical in ABP studies, to take a reading at 30-minute intervals (O'Connor, O'Connor, White, & Bundred, 2000). Participants were blinded to the values of their BP readings during monitoring. Participants were given a diary and asked to record their activity level (1 = *no activity* to 4 = *strenuous activity*) immediately after each monitor reading. Prior to data analysis, the ABP readings for each participant were screened for outlying values and artefactual readings. However, no outliers or potentially artefactual readings were identified.

### Procedure

The study received ethical approval from the University Department ethics committee. Students who responded to advertisements were randomized to a writing condition and emailed study information. Eligible students who wished to participate then scheduled a baseline laboratory session during which written consent was obtained, height, weight and resting BP were measured and baseline questionnaire measures (e.g. RRS) were administered. At the end of this session, participants were given instructions and materials for the writing intervention. Participants were asked to write at home in private, continuously for 20 minutes, once a day, on 3 consecutive days. Participants dated and sealed their essays after each writing session, and then returned all three to the university. Following this, dates for ABP monitoring were scheduled for 2 weeks and 14 weeks post-writing.

### Data analysis

These data were analyzed utilizing hierarchical linear modelling (HLM) (Raudenbush, Bryk, Cheong, & Congdon, 2004) using HLM6. They contained a two-level hierarchical structure, Level 1 being the within-person variation (i.e. 30-minute ABP readings throughout the day, activity level at time of each BP reading) and Level 2 being the between-person variability (i.e. writing condition and brooding score). Activity level, parental hypertension, baseline BP, age and BMI were controlled for in each of the models given their known effects on cardiovascular outcomes. The Level 1 activity level variable was group mean centred (Nezlek, 2001). The continuous Level 2 variables (e.g. brooding) were grand mean centred and the dichotomous variables (e.g. condition) uncentred (Nezlek, 2001). In order to explore the predicted interaction between writing condition and brooding a Level 2 multiplicative interaction term was computed and entered into each model. The analyses were conducted in two stages. First, the main effects of writing condition, brooding and the condition × brooding interaction were modelled (while controlling for parental hypertension, age, BMI and baseline BP levels) in terms of ABP levels at 2 weeks and then separately at 14 weeks post-writing intervention. Second, significant condition by brooding interactions were decomposed using the procedures recommended by Preacher, Curran, and Bauer (2006).

## Results

### Descriptive statistics

The mean levels of systolic blood pressure (SBP) and DBP at resting (109.12 ± 9.13 mmHg, 64.81 ± 7.05 mmHg, respectively) and during the ambulatory monitoring (117.92 ± 12.18 mmHg, 72.54 ± 9.80 mmHg, respectively) were within normal healthy ranges (see Table 1). Participants in the WED and control writing conditions did not differ significantly on any of the main study variables thus confirming baseline equivalence and successful randomization. Manipulation checks were conducted to determine intervention fidelity. Essays were read and analysed using the Linguistic Inquiry and Word Count analysis program (Pennebaker, Francis, & Booth, 2001). Multivariate ANOVA revealed that there were significantly more negative emotion, insight and causation words used in the WED condition essays compared to the control condition essays, *F*(3, 48) = 159.55, *p *< .001.

**Table 1.** Descriptive statistics for final sample and main study variables (*n *= 52).

	Mean	SD
Age	18.37	0.66
BMI	22.30	2.79
% Caucasian	85.1%	–
% Parental hypertension	27.5%	–
Baseline SBP	109.12	9.13
Baseline DBP	64.81	7.05
Ambulatory SBP	117.92	12.18
Ambulatory DBP	72.54	9.80
Brooding score	10.98	2.95
Reflection score	11.39	3.53

Note: SBP = systolic blood pressure and DBP = diastolic blood pressure; BMI = body mass index.

### Effects of brooding and writing condition on ABP

The results of the HLM analysis showed that in terms of the control variables, higher baseline SBP, activity level and having at least one parent with a history of hypertension were associated with greater SBP. However, importantly with these variables controlled there was a marginal effect of writing condition on SBP levels two weeks post-writing (*p* = 0.052), with greater levels of SBP following WED writing compared to control writing (Table 2). There was also evidence of a condition by brooding interaction with *p* again approaching conventional significance (*p* = 0.052). The pattern of results was similar and stronger for DBP. The effects of both writing condition and the writing condition by brooding interaction were statistically significant. Again greater levels of DBP were observed following the WED writing compared to the control writing condition at two weeks follow-up (Table 2).

**Table 2.** Effects of writing condition and brooding on ABP at 2 and 14 weeks follow-up (controlling for parental hypertension, activity levels, age and BMI).

	Two weeks follow-up				Fourteen weeks follow-up			
HLM effect	Symbol	Coeff.	SE	*p*	Symbol	Coeff.	SE	*p*
*Intercept SBP level*	*β*_00_	115.53	2.66	<.001	*β*_00_	114.43	2.454	<.001
Baseline SBP level	*β*_01_	0.62	0.11	<.001	*β*_01_	0.54	0.11	<.001
Parental hypertension	*β*_02_	−3.83	1.56	<.05	*β*_02_	−4.43	1.78	<.05
Writing condition	*β*_03_	10.40	5.21	.052	*β*_03_	6.95	5.68	.23
Age	*β*_04_	1.82	1.09	.10	*β*_04_	1.85	1.29	.19
BMI	*β*_05_	0.30	0.29	.31	*β*_05_	−0.07	0.28	.74
Brooding	*β*_06_	−0.09	0.30	.76	*β*_06_	−0.08	0.31	.73
Condition × brooding	*β*_07_	−0.91	0.45	.052	*β*_07_	−0.37	0.45	.43
*Level 1 slope*								
Activity level – SBP level	*β*_10_	4.51	0.58	<.001	*β*_10_	4.51	0.58	<.001
*Intercept DBP level*	*β*_00_	69.10	2.26	<.001	*β*_00_	68.89	1.90	<.001
Baseline DBP	*β*_01_	0.43	0.10	<.001	*β*_01_	0.28	0.10	<.01
Parental hypertension	*β*_02_	−2.52	1.19	<.05	*β*_02_	−3.17	1.44	<.05
Writing condition	*β*_03_	11.12	4.58	<.05	*β*_03_	7.03	4.16	.11
Age	*β*_04_	1.22	0.79	.13	*β*_04_	2.30	1.09	<.05
BMI	*β*_05_	0.05	0.15	.77	*β*_05_	0.03	0.22	.90
Brooding	*β*_06_	0.16	0.26	.54	*β*_06_	0.37	0.25	.14
Condition × brooding	*β*_07_	−0.96	0.42	<.05	*β*_07_	−0.56	0.35	.12
*Level 1 slope*								
Activity level – DBP level	*β*_10_	3.58	0.48	<.001	*β*_10_	4.71	0.67	<.001

Note: HLM = hierarchical linear modelling; Symbol = hierarchical multivariate linear modelling symbol; Coeff. = unstandardized coefficient; SE = standard error; SBP = systolic blood pressure; DBP = diastolic blood pressure; BMI = body mass index.

**Figure 1.**
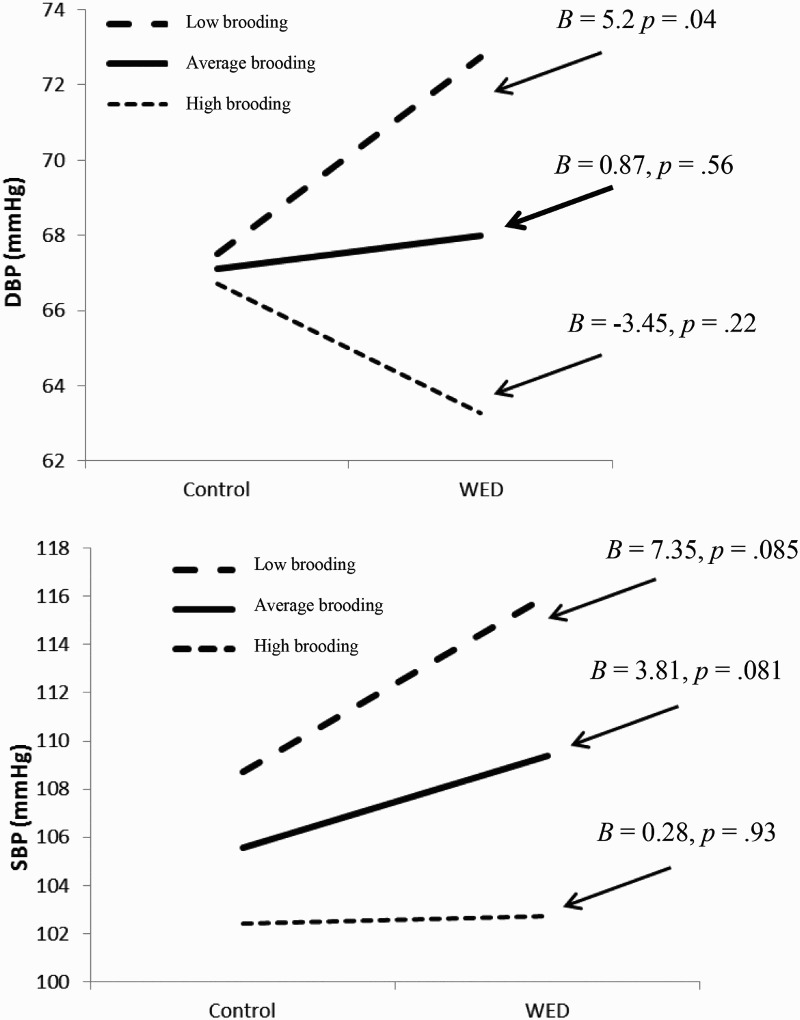
Interactions of brooding by condition on DBP and SBP at week 2 follow-up. Low brooding = 1.5 SDs below the mean. Average brooding = mean. High brooding = 1.5 SDs above the mean. WED = written emotional disclosure.

These interactions were decomposed using the simple slopes procedures developed by Preacher et al. (2006) for multi-level modelling and are shown in Figure 1. Considering the top panel, for DBP, the results show that for those low in brooding there was a significant effect of condition (*B* = 5.20, *p* = .04), such that higher DBP was observed in low brooders two weeks following the intervention compared to brooders in the control condition. In the high brooders, lower DBP was exhibited in the WED condition compared to the control condition, however this difference was not statistically significant (*B* = −3.45, *p* = 22). Moreover, within the WED condition there was a significant effect for brooding (*B *= −1.06, *p *= .0004) such that those higher in brooding had significantly lower DBP compared to those lower in brooding. There were no significant effects of brooding in the control condition.

The same pattern is observed for SBP. For low- (*B *= 7.35, *p *= .085) and mean-level (*B *= 3.81, *p *= .081) brooders, there was an effect of condition that approached significance such that higher SBP was observed in low brooders two weeks following the intervention compared to low brooders in the control condition. Again within the WED condition there was an effect of brooding (*B *= −1.5 *p *= .0011) such that participants high in brooding exhibited significantly lower SBP compared to those low in brooding. There were no significant effects of brooding in the control condition.

The same analyses were performed at 14 weeks follow-up and revealed that only the control variables were significantly associated with daily BP levels indicating no main or interactive effects of brooding or condition.

In terms of percentage of variance explained, for SBP at 2 weeks, the Level 2 variables accounted for 33.5% of the variance in SBP. For DBP at 2 weeks they explained 25% of the variance in DBP. At 14 weeks, the Level 2 variables explained 37% and 31.8% of the variance in SBP and DBP, respectively.

### Effects of reflection and writing condition on ABP

The same analytical strategy was utilized to explore the main and interactive effects of reflection. There were no significant main or interactive effects either at 2 weeks or 14 weeks follow-up (data not shown).

## Discussion

Three main findings emerged from the current study. First, a brooding ruminative response tendency moderated the effects of writing condition, such that higher ABP levels at follow-up were observed in low brooders in the WED condition compared to low brooders in the control condition. Second, within the WED condition, the lowest ABP levels were exhibited by participants high in brooding. Third, WED led to short-lived increases in ABP which disappeared in the medium term.

In the current study, the moderating effects of brooding are particularly interesting as they suggest that WED, in the short term at least, has the capacity to confer meaningful changes in daily cardiovascular outcomes for individuals with a lower brooding ruminative response style. Specifically, these findings show that WED significantly increases DBP in individuals who score low in brooding with the effect being marginal for SBP, yet they also tentatively (and non-significantly) suggest that WED may be beneficial for high brooders. Inspection of Figure 1 shows that ABP levels are lower for high brooders in the WED condition compared to the controls (especially for DBP). We are mindful that the latter finding is non-significant, and therefore should be treated with caution; however, it is possible that this effect may have been statistically significant in a larger sample. Indeed, it is important to note that, in the WED condition, the lowest levels of SBP and DBP were observed in the high brooding group.

As outlined earlier, we found that WED was associated with increases in ABP (especially DBP) for participants low in brooding. However, importantly, these increases returned to baseline by the second follow-up assessment and were within the normal healthy range. While at first glance this may be interpreted as a detrimental effect, however, the changes in ABP are not clinically significant with the highest levels for SBP and DBP levels substantially below the range for high BP or hypertension (i.e. 140/90 mmHg, see Figure 1). Instead, these short-term increases are likely to reflect the processes underlying the participant (re)engaging with the stressful/traumatic event and memories. For example, Schwartz et al. (2000) showed that asking participants to recall an anger-provoking event led to increases in BP. They also demonstrated that having intrusive thoughts about the event hampered the participants’ BP recovery. These authors and others (Davidson et al., 2002; O'Connor, Walker, Hendrickx, Talbot, & Schaefer, 2013) suggest that these elevations in BP and intrusive thoughts are likely to reflect unsuccessful cognitive integration of the stressful experiences. Moreover, it has been argued that a longer initial follow-up may be required in order for positive improvements to emerge (O'Connor & Ashley, 2008; Wetherell et al., 2005). For example, in relation to BP levels, Beckwith McGuire et al. (2005) observed beneficial effects of WED four weeks post-writing. With this in mind, future research ought to attempt to identify the timeline along which effects of WED operate by including multiple follow-up assessments in the immediate (i.e. last day of writing), short (i.e. at two weeks), medium (i.e. one month) and longer term (i.e. six months).

While we did not generate directional hypotheses, the Sloan et al. (2008) findings alone would have suggested that those high in brooding ought to exhibit reduced ABP in the WED condition. Our results revealed that those low in brooding showed significantly increased ABP in the WED condition while there were no statistically significant reductions in high brooders (although, this group exhibited the lowest ABP levels in the WED condition but did not differ significantly from their control counterparts). One potential explanation that might account for these findings relates to familiarity. It is possible that exposure to WED does not mark a context that is unfamiliar to those high in brooding tendency. Indeed high brooders may frequently find themselves in such contexts (i.e. thinking about past stressful/traumatic encounters), this being the case the lack of clear and strong effects of WED on ABP may reflect familiarity. Low brooders on the other hand will probably rarely encounter context requiring reflecting on past emotions and this lack of familiarity leads to higher ABP. An alternative explanation might be that the WED intervention simply is not beneficial for individuals low in brooding. Nevertheless, these competing explanations notwithstanding, the current results are also important as they suggest that individual differences in brooding may account for (some of) the mixed and inconsistent findings in past WED research (cf. Frattaroli, 2006; O'Connor et al., 2013; Smyth & Pennebaker, 2008).

These findings do not necessarily have to be seen as conflicting with those of Sloan et al. (2008). Sloan and colleagues showed beneficial effects of WED on emotions and cognitions in brooders; our results show that at a physiological level WED is not detrimental and indeed, in a larger sample, may yield positive effects on ABP. Therefore, to our mind, it is incumbent on researchers to build upon this exploratory study and to investigate further the potential moderating role of the brooding ruminative response tendency in larger healthy and clinical samples.

We recognize that the current study has a number of shortcomings and limitations that require further comment. The participants under study were young, healthy and normotensive; therefore, the results may not be generalizable to older participants who have chronic health conditions or who have experienced chronic stress or trauma over many years. Nevertheless, future research ought to attempt to replicate the current findings utilizing ecological momentary assessment and diary methods to explore the mechanisms of action associated with the effects of WED and to measure different physiological markers of the disclosure process (O'Connor et al., 2013; Segerstrom & O'Connor, 2012).

In conclusion, the current study showed that brooding moderated the effects of writing condition, such that higher ABP levels at follow-up were observed in low brooders in the WED condition compared to low brooders in the control condition. However, within the WED condition, the lowest ABP levels were exhibited by participants high in brooding. The findings also indicated that WED led to short-lived increases in ABP which disappeared in the medium term. Individual differences in brooding may account for (some of) the mixed and inconsistent findings in past WED research.

## Note

1. In multi-level models there are sample sizes associated with each level in the model. Determining power is, therefore, much more complicated than in the case of ordinary least squares fixed effects regression and it is argued that the sample for estimating power is primarily determined by the level in the analyses where the main predictors are located (Maas & Hox, 2005; Snijders, 2005). For cross-level interactions, the focus of this study, the picture is more complicated and power depends on the number of Level 2 units, the number of Level 1 assessments and the SD of the slope estimate (Mathieu, Aguinis, Culpepper, & Chen, 2012). Mathieu et al. (2012) suggest that to detect a cross-level interaction “ … power would reach 80% for relatively large Level 2 samples (i.e. 115) and SD of slope of approximately 0.5” (p. 958)”. However, for Level 1 they suggest that “ … for relatively large average Level 1 sample sizes (i.e., 18), power exceeded 50% for SD of slopes at 0.17, although it would not reach 80% unless the SD of slopes exceeded 0.3” (p. 958). In our data, the average Level 1 sample size was 24 observations per person at T1 and 23.7 at T2. Importantly, these also further show that for “ … relatively large average Level 1 samples sizes (i.e., ≥ 18) afford power > 60% with Level 2 samples as small as 25 and surpass power of 80% with Level 2 samples of 35” (p. 958). Thus given our number of Level 1 and Level 2 observations, we feel confident that our sample is relatively well powered.
